# Health Tract, No. 259

**Published:** 1866-11

**Authors:** 


HEALTH TRACT, No. 259.

### PURE AIR.

A beneficent providence has arranged that while the air we breathe gives us life by purifying the blood and imparting vital heat, its unfitness for these purposes is instantly determined by such disagreeable impressions on the senses, that we instinctively cease breathing, or hasten from the contaminated spot. Then again, from her bountiful stores, nature has provided substances which purify filthy localities and remove the nauseous smells. Some of these substances destroy the disagreeable odors but do not arrest decay, hence those odors would return constantly, as long as any of the substance remained which was the source of the evil. Chlorine removes a bad smell from putrid meat, but it will return in a few hours. A London Chemist has ascertained the fact that if a piece of fresh meat is coated with a substance distilled from coal and mixed with sulphurous acid, called carbolic acid, it will prevent the meat from decaying, and that such meat after being kept two or three months, is, if cooked, as sweet and fresh as meat just purchased from the butcher's stand; hence chlorine, deodorizes, that is only takes away the bad smell for the present, but decay still goes on. Carbolic acid not only destroys the odor but prevents decay, arrests it, and thus is a deodorizer and disinfectant combined; it takes from a substance its polluting character, its power to make sick, to communicate disease. If further and more careful investigations and experiments confirm the statements made by Mr. Crooks he is justly entitled to be named among the benefactors of the age. The practical lesson to be impressed on the mind by these statements is, that a deodorizer does no more than take away the ill odor of substance or locality, temporally; a disinfectant not only destroys that odor but prevents its return, by changing that condition of the substance from which the odor came, in a manner which does not allow the process to go on which gave rise to the odor. A disinfectant also takes from a thing its power to cause disease. A contagious disease is that which is caused by actual contact, and cannot be communicated in any other way, as glanders in horses, and syphilis in men; infection is that which may be communicated by touching or handling the clothing as itch, plague, measles, small pox; the air of a room in which these diseases are, can communicate the disease, hence that air is infectious, that is, makes into, fixes, implants, thrusts into the body, the disease with which it is loaded. A real disinfectant deprives the air and the clothing of that power. It seems that carbolic acid is the most perfect deodorizer and disinfectant yet made known to man.

